# Upstream Disaster Management to Support People Experiencing Homelessness

**DOI:** 10.1371/currents.dis.95f6b76789ce910bae08b6dc1f252c7d

**Published:** 2015-08-18

**Authors:** Madura Sundareswaran, Andrea Ghazzawi, Tracey L. O'Sullivan

**Affiliations:** University of Alberta; University of Ottowa; Interdisciplinary School of Health Sciences and Telfer School of Management, University of Ottawa, Ottawa, Ontario, Canada

## Abstract

The unique context of day-to-day living for people who are chronically homeless or living with housing insecurity puts them at high risk during community disasters. The impacts of extreme events, such as flooding, storms, riots, and other sources of community disruption, underscore the importance of preparedness efforts and fostering community resilience. This study is part of larger initiative focused on enhancing resilience and preparedness among high risk populations. The purpose of this study was to explore critical issues and strategies to promote resilience and disaster preparedness among people who are homeless in Canada. A sample of interviews (n=21) from key informants across Canada was analyzed to explore existing programs and supports for homeless populations. The data was selected from a larger sample of (n=43) interviews focused on programs and supports for people who are at heightened risk for negative impacts during disasters. Qualitative content analysis was used to extract emergent themes and develop a model of multi-level collaboration to support disaster resilience among people who are homeless. The results indicate there is a need for more upstream continuity planning, collaboration and communication between the emergency management sector and community service organizations that support people who are homeless. Prioritization and investment in the social determinants of health and community supports is necessary to promote resilience among this high-risk population. The findings from this study highlight the importance of acknowledging community support organizations as assets in disaster preparedness. Day-to-day resilience is an ongoing theme for people who are chronically homeless or living with housing insecurity. Upstream investment to build adaptive capacity and collaborate with community organizations is an important strategy to enhance community resilience.

## Introduction

Homelessness is a growing problem across North America. In Canada, while estimates vary, there may be up to 150 000 people each night who turn to emergency shelters 1. Approximately 733,275 households (18% of those who are renting) are at risk of homelessness at any time, due to housing affordability issues, with more than 50% of their income being put toward rent 2. In recent years, as more people have experienced housing insecurity, there has been increased demand for governments, non-profit organizations, and communities to respond to the complex needs of this population 3 ^,^ 4 ^,^ 5. Inconsistent definitions for ‘homelessness’ and difficulties identifying this population (due to transient lifestyles and intermittent use of community services), have created distinct challenges in responding to needs 6.

Immediate and extended support services such as shelters, soup kitchens, counseling, and assistance with employment skills and re-integration into the community are common types of supports for people experiencing homeless 7 ^,^ 8 ^,^ 9. Despite the availability of these programs, social isolation, marginalization, lack of resources and protection, inadequate healthcare, and overcrowded spaces remain challenges for people who are homeless, exacerbating their risk during disasters 10 ^,^ 11. This heightened risk was evident in Canada during Hurricane Juan, and the outbreaks of Severe Acute Respiratory Syndrome (SARS) and H1N1, underscoring the need to focus on upstream supports that address social determinants of health, to enhance adaptive capacity among populations who may be unable to protect themselves during disasters 11 ^,^ 12.

Disasters can also be the cause of homeslessness. For example, Hurricane Katrina destroyed 60% of the housing in New Orleans; leaving a vast number of people reliant on local supports and services, including housing 13. The impacts of the 2015 earthquakes in Nepal are reminders of how home security can change instantaneously during an extreme event, leaving many people without secure, stable shelter.

Resilience has been described as the capacity to absorb and adapt to the impacts of an adverse event within the social and physical environment 14 ^,^ 15. Homelessness demands people demonstrate resilience on a daily basis, when living in a context often characterized by crowded living conditions, substance abuse, people coping with mental illness, violence, and difficulties accessing care for chronic medical conditions 6. These daily living conditions create a delicate social fabric, which presents heightened risk during community disasters; however empirical literature exploring this risk and strategies to manage it has been limited 11 ^,^ 16.

Donaldson et al. 16 explored the experiences of persons who were homeless during the 2002 Washington sniper shootings. They reported fear for their safety, anger and sadness, changes to lifestyle behaviours, and the need to employ a range of coping strategies. The authors recommended the use of an emergency management lens to identify the specific needs of people who are homeless, acknowledging that those needs may differ from those of the general population. It is also important to acknowledge the strengths/assets of this population, which is made up of people with extensive experience managing uncertainty and stress, as this could have protective effects in a disaster context 17.

The needs of people who are homeless was discussed by Leung et al.^11^ in the context of lessons from SARS. They stressed the importance of collaborative planning and identifying the capacity of existing shelters and community service providers. Communication and collaboration were important themes, paticularly for developing a comprehensive strategy around protocols for isolation, quarantine, and resource allocation in the management of future outbreaks.

In this study, we set out to identify types of community assets and supports for people who are homeless which can promote resilience and preparedness for disasters. This study is part of <blinded project> which is a multi-community initiative focusing on resilience and preparedness among high-risk populations.

## Method

Professionals and volunteers working in emergency management, health and social services were recruited to participate in telephone interviews using snowball sampling strategies. All participants signed consent forms approved by the university research ethics committee before taking part in a 30 minute audio-recorded semi-structured phone interview. Questions included: “Can you tell us about any programs you are aware of that are designed to enhance resilience and/or preparedness for high risk populations?” and “What do you feel are the successful ingredients for a resilience/preparedness intervention for high risk populations?” Each interview was transcribed verbatim and checked for accuracy. Among the total (n=43) key informants, (n=21) mentioned people who are homeless in their interviews. This sub-set of interview transcripts was used as the dataset for this study. A coding grid was developed inductively, and all transcripts were double-coded by the first two authors, to enhance rigour. Using a consensus approach, all three authors conducted thematic analysis to identify emergent themes and develop the conceptual model (Figure 1).

## Results

The key informants who participated in the study were (n=4) volunteers and (n=13) professionals in disaster management, and (n=4) professionals in social services, from across Canada. The six emergent themes focused on identification of homeless populations, recognition of functional capabilities and limitations as central issues for disaster planning, the need to reduce marginalization, and the importance of continuity planning and accessibility of informational resources. Lack of resources was identified as a critical barrier to sustainability and supporting people who are chronically homeless or experiencing housing insecurity, while the need for inter-organizational collaboration and prioritization of homeless populations in disaster planning were highlighted as important areas for intervention and policy development.

**Emergent Themes**

1. A forgotten population in disaster preparedness planning

The key informants described how people who are chronically homeless or those who experience housing insecurity are often overlooked in disaster planning *“...unfortunately at-risk populations don’t represent the majority of the people...sometimes [people who are homeless] pushed to the side when we are dealing with instantaneous situations where things just need to be put in place.”* Some community disaster plans are structured for the general population, without specific contingencies for people who do not have secure housing. As explained by one participant, community plans are "built for 80-90% of the population, not the 10% that is vulnerable.”

2. Appropriate and accessible channels for information exchange must be in place

Many preparedness programs target the general population and provide information through channels such as the internet and television, which are often not accessible for people who are homeless or experiencing housing insecurity. Community associations and other support services who are in regular contact with at-risk populations represent important hubs for the dissemination of important information before, during and after a community disaster. As expressed by one key informant, *“there is an issue of access to information. [People who are homeless] can’t necessarily get information from CBC… information should be made available to soup kitchens...”*.

3. Prioritize continuity of operations planning for community associations

Lack of continuity planning by community support organizations is a gap that threatens adaptive capacity. The participants expressed concern about where supports for people who are homeless would come from if existing programs and services had to close, due to lack of staff and volunteers during a community disaster. Community organizations are often lean in terms of available human resources and time to dedicate to continity of operations planning, however that investment in upstream continuity planning contributes to adaptive downstream response. *“[People who are homeless would be] more at risk because the services they rely on, on a daily basis, would be minimized or compromised during a pandemic…we were estimating that the [percentage of] staff off could be up to 30% in terms of people leaving sick or choosing not to come to work. And it’s losing those services that really create disparities, and it makes people more at risk...”*

4. Stable funding is needed to support program sustainability

The key informants who worked in shelters expressed concern about policies and gaps in governmental support (such as funding schemes) which create a culture of uncertainty for program planning, and limit organizational capacity to plan for continuity of operations in the event of a community disaster. For example, one person described an issue with stocking emergency supplies for pandemic: *“We were lucky that we accessed some society money and bought some good masks, and some gloves and you know tried to think of what would happen if we had a bunch of sick people ... we were able to do that which was really good, but not every place could.”* Another key informant expressed frustration with planning for program sustainability: *“So, if we had [funding],…we would promote that, go and do it. But… you gather the information and then the funding is gone. So it’s hard to keep up, keep it going.”*

5. Disaster preparedness often takes a back seat to other priorities

Many key informants stated that disaster preparedness is not a priority when shelter and food insecurities are daily challenges. Other basic needs must be met before thinking about preparedness for uncertain events. An important aspect of this is the lack of available resources for upstream disaster management. *“… peoples’ lives are kind of fraught with personal disaster all the time down here. I don’t know if people necessarily would, it’d be like one more thing... [From their perspective], right now I need to eat I need to get into some shelter I don’t need to think about, you know a tornado blowing through here.”*

Preparedness is influenced by clustering of the social determinants of health (eg. income and housing insecurity, employability, social and physical environments). Prioritization of investments in interventions that enhance asset profiles of people at risk can enhance adaptive capacity for downstream response when a community disaster occurs. Investment in collaborative activities to develop, implement and evaluate upstream interventions is an important strategy to enhance preparedness.

6. Asset literacy is an important aspect of multi-sector collaborative relationships

Front line community support workers have important expertise and connections with people who are homeless or experiencing housing insecurity. Asset literacy refers to not only identifying the assets within the community organizations, but recognizing how those assets can contribute in a disaster, ensuring people and organizations know how to mobilize available assets, and having the self-efficacy and motivation to activate assets across multiple levels.^17^ As expressed by one key informant: *“So the city has a great disaster plan but it’s kind of a bit of a secret… unless you go online you can’t find it. But it would be nice if they had some contact with downtown core agencies, because really the agencies are the ones who will, through their programs, get their word out to the residents of the downtown core. So you need them as a link in the chain.”*

Collaboration between emergency managers and community level organizations is essential to enhance resilience among people who are homeless. It is important to leverage the relationships community organizations have with people who are homeless to maximize information exchange and supports for this often hard-to-reach population. These relationships are important assets to support adaptive capacity.

**Emergent Model**

Figure 1 depicts a model of multi-level collaboration to enhance disaster resilience among people who are homeless, integrating the emergent themes from this study with existing literature, with particular emphasis on existing services and supports, and links with the emergency management sector and other organizations across multiple social-ecological levels. The results underscore the importance of inter-organizational collaboration, and the need to leverage key assets from each organization and individual, to enhance community capacity to adapt to changing context. Interrelated factors include collaborative planning, sharing of knowledge and resources, and communication, all of which promote asset literacy and creative problem-solving for a complex issue.

**A Model of Multi-level Collaboration to Enhance Disaster Resilience for People Who Are Homeless:**
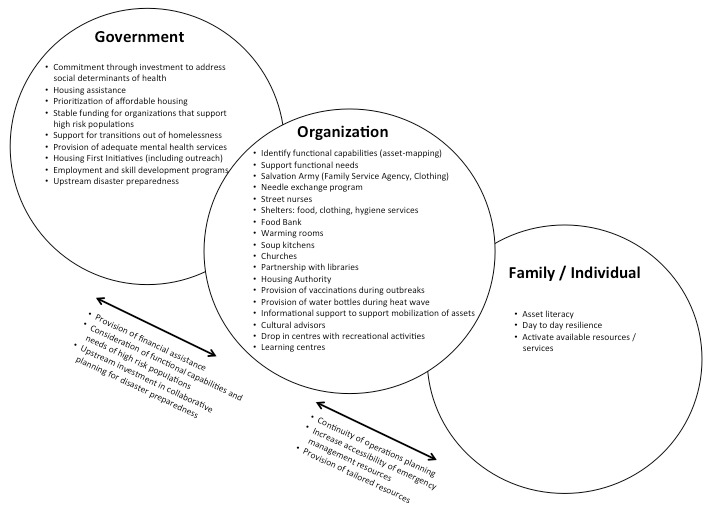

## Discussion

Depending on the type and complexity of a given disaster, a large segment of the population may be at-risk 18. For people who are homeless or who are experiencing housing insecurity, the combination of a disaster plus the pre-existing challenges of day-to-day living exacerbates risk. Contingency planning for this segment of the population is necessary for all municipalities and essential service organizations 12 ^,^ 13 ^,^ 16. Although there is literature focused on people who have become homeless as a result of a disaster, there is limited empirical literature discussing issues related to disaster preparedness among people who are homeless or experiencing housing insecurity before an adverse event occurs 11 ^,^ 16. The current study was conducted to explore critical issues related to the provision of support for people who are homeless, and to identify strategies to promote resilience and disaster preparedness. The emergent themes emphasize the importance of recognizing functional capabilities in combination with needs, and to prioritize upstream investments such as continuity of operations planning and investment of long-term financial resources to address social determinants of health.

The key informants in this study identified addiction, lack of access to informational resources, and transiency, as factors contributing to heightened risk during disasters. This is consistent with literature related to daily challenges for people who are chronically homeless or those who continually experience housing insecurity 10. If services such as emergency housing and soup kitchens are disrupted due to a community disaster, people who are homeless may not have their basic needs met. For people with additional, complex functional limitations, such as mental illness, and/or addictions, additional supports will be required to ensure affected individuals can manage at an emergency shelter set up for the general public. Shelter volunteers may not be trained or prepared to assist people with specific types of functional limitations, therefore contingency planning to ensure adequate staff and volunteers are available, becomes a necessity 13 ^,^ 18.

Programs and services for people who are homeless are typically provided at the community level 4. As our findings indicate, this places tremendous responsibility on community organizations such as shelters, food banks and soup kitchens. In addition, the demand for support and services escalates as the number of people who are displaced increases; particularly during mass evacuation as part of the disaster response 13. Disaster preparedness activities should focus on identifying the assets of the people who are homeless and the community support organizations through activities such as asset-mapping; as well as invest time in building or strengthening relationships across multiple levels of government and the private sector. Our findings are supported in the literature, as plans for more effective communication 19, and ensuring the sustainability of services during disasters can be implemented by enhancing partnerships between service providers and those whose role it is to manage a disaster 11 ^,^ 17.

An important upstream strategy to promote adaptive capacity among people who are homeless or experiencing housing insecurity, is to address the determinants of homelessness. This intervention strategy is represented in Figure 1, with the private sector and multiple levels of government collaborating as key partners tackling this complex issue. For example, financial support for community programs which focus on prevention and transition out of homelessness is essential (eg. housing programs and counseling) 2. Gaetz 4 described how previously the lack of a National Housing Strategy, combined with shifts in government policies and reduced support for low-income families, has led to increased prevalence of homelessness. An important advance in the last year has been the introduction of Housing First legislation across Canada, which is a system-level intervention to address housing availability and accessibility among people who are chronically or episodically homeless 2. These settings-based interventions are needed to reduce inequities and enhance adaptive capacity by addressing macro-level influences on social determinants of health 20.

The National Homelessness Information System is another important initiative in Canada, providing demographic information about who is homeless, the reasons they are experiencing homelessness or housing insecurity, their needs, and which interventions are effective in addressing those needs 21. The following quotation from Gaetz et al. 2 emphasizes the importance of this initiative and how it prompts social innovation via systems-level thinking about homelessness; reflecting on how current programs are addressing the issue and how collaborative practice can address stresses within the system. *“In an age of ‘Big Data’ and advanced technology, we should be able to know in real time exactly how many Canadians are homeless, who they are and whether the interventions they receive are effective. We should be able to respond to their needs with a coordinated system of care that is simple for clients to navigate. And we should be able target resources in the homeless system to those who need it the most. It should also be possible to reduce the administrative burden faced by front-line agencies, by streamlining reporting to multiple funders and referrals to partner agencies”* 2 (p.19).

The key informants in our study discussed the importance of engaging people from within community organizations and recognizing the expertise available within programs and services at the community level to promote disaster preparedness. This strategy employs an assets-based approach 22, which focuses on the mobilization of existing assets within an organization or community to enhance adaptive capacity. Situational awareness is an example of this type of asset, which can be enhanced by inter-organizational collaboration in disaster planning, and sharing the knowledge of critical issues to promote asset literacy 17.

Several participants stated that the information provided to them regarding disaster planning may be inaccessible to persons who are homeless due to difficulties in comprehension and accessibility to media. Through inter-organizational collaboration, people who are homeless or experiencing housing insecurity may be better informed, provided the information is tailored and relevant to their needs. One consideration is to account for different literacy levels or sensory limitations 23. Tailoring of services and supports may be more easily implemented by community organizations with established relationships with those who they support on a day-to-day basis, provided there is support for these organizations to meet these additional demands 8 ^,^ 18.

## Conclusions

People who are homeless or experiencing housing insecurity face tremendous challenges in day-to-day living; basic needs must be prioritized over disaster preparedness. Upstream strategies that address social determinants of health can contribute to adaptive capacity across individual, organizational and community levels. Creating opportunities for inter-organizational collaboration between all levels of government and organizations providing front line supports for people who are chronically or episodically homeless is an important investment to enhance adaptive capacity. Continuity of operations planning for community organizations is an important practice to ensure essential support services remain functional. However, stable financial support is needed to ensure program sustainability.

## Competing Interests

The authors have declared that no competing interests exist.
